# Prolongation of allograft survival by passenger donor regulatory T cells

**DOI:** 10.1111/ajt.15212

**Published:** 2019-02-05

**Authors:** Ines G. Harper, Olivera Gjorgjimajkoska, Jacqueline H. Y. Siu, Jasvir Parmar, Arend Mulder, Frans H. J. Claas, Sarah A. Hosgood, Michael L. Nicholson, Reza Motallebzadeh, Gavin J. Pettigrew

**Affiliations:** ^1^ Department of Surgery School of Clinical Medicine University of Cambridge Cambridge UK; ^2^ Department of Cardiothoracic Transplantation Papworth Hospital Cambridge UK; ^3^ Department of Immunohaematology and Blood Transfusion Leiden University Medical Center Leiden The Netherlands; ^4^ Centre for Surgical Innovation, Organ Repair & Transplantation University College London London UK; ^5^ Centre for Transplantation, Department of Renal Medicine University College London London UK; ^6^ Institute of Immunity and Transplantation University College London London UK

**Keywords:** basic (laboratory) research/science, cellular biology, immune regulation, immunobiology, lymphocyte biology, organ transplantation in general, T cell biology, tolerance: experimental, tolerance: mechanisms, translational research/science

## Abstract

Tissue resident lymphocytes are present within many organs, and are presumably transferred at transplantation, but their impact on host immunity is unclear. Here, we examine whether transferred donor natural regulatory CD4 T cells (nT‐regs) inhibit host alloimmunity and prolong allograft survival. Transfer of donor‐strain lymphocytes was first assessed by identifying circulating donor‐derived CD4 T cells in 21 consecutive human lung transplant recipients, with 3 patterns of chimerism apparent: transient, intermediate, and persistent (detectable for up to 6 weeks, 6 months, and beyond 1 year, respectively). The potential for transfer of donor nT‐regs was then confirmed by analysis of leukocyte filters recovered from ex vivo normothermic perfusion circuits of human kidneys retrieved for transplantation. Finally, in a murine model of cardiac allograft vasculopathy, depletion of donor CD4 nT‐regs before organ recovery resulted in markedly accelerated heart allograft rejection and augmented host effector antibody responses. Conversely, adoptive transfer or purified donor‐strain nT‐regs inhibited host humoral immunity and prolonged allograft survival, and more effectively so than following administration of recipient nT‐regs. In summary, following transplantation, passenger donor‐strain nT‐regs can inhibit host adaptive immune responses and prolong allograft survival. Isolated donor‐derived nT‐regs may hold potential as a cellular therapy to improve transplant outcomes.

**Abbreviations**
ANOVA: analysis of variance
APC: allophycocyanin
CAV: chronic allograft vasculopathy
DMSO: dimethyl sulfoxide
EDTA: ethylenediaminetetraacetic acid
EVG: elastin van Gieson
FCS: fetal calf serum
MST: median survival time
nT‐regs: natural regulatory CD4 T cells
PBMCs: peripheral blood mononuclear cells
PE: phycoerythrin
PBS: phosphate‐buffered saline
RT‐PCR: real time‐polymerase chain reaction
SD: standard deviation

## 1 INTRODUCTION

Although still considered a novel technology, ex vivo perfusion of recovered organs from deceased donors is likely to become widely adopted in the near future.^1^, ^2^ Ex vivo perfusion offers the potential to assess the viability of organs before transplantation, and to extend the acceptable period between recovery and implantation. It may also enable targeting of the isolated organs with specific therapies aimed at prolonging allograft survival.^3^ One particular focus of such strategies is likely to be donor‐derived T cell populations (naïve or memory) that are resident within the graft.^4^, ^5^

We have recently reported that passenger T cells are present within human donor organs recovered for transplantation and, using murine transplant models, have demonstrated that donor T effector cells can augment host alloimmune responses directed against the allograft.^6^ Thus, although seemingly counterintuitive, these passenger lymphocytes contribute to rejection of the organ. Here, we examine whether donor‐derived natural regulatory CD4 T cells (nT‐regs) can, conversely, prolong allograft survival.

## 2 MATERIALS AND METHODS

### 2.1 Identification of circulating donor CD4 T lymphocytes in human lung transplant recipients

Following adult deceased‐donor lung or heart plus lung transplantation, blood from consenting recipients was sampled at predetermined time points (initially weekly for the first 2 months after transplantation, and monthly/bimonthly thereafter) and donor CD4 T lymphocytes was identified by flow cytometry, on the basis of the expression of MHC alloantigen. Briefly, peripheral blood mononuclear cells (PBMCs) were labeled with anti‐CD3‐FITC (fluorescein isothiocyanate, clone HIT3a) and anti‐CD4 PE (phycoerythrin, clone RPA‐T4) monoclonal antibodies (both BD Biosciences, Oxford, UK) and with the relevant MHC class I HLA‐specific biotinylated antibody that were selected to bind exclusively to donor (but not recipient) HLA class I MHC alloantigen (see Table S1; kindly gifted by Prof. Frans Claas, Leiden University Medical Center, Leiden, the Netherlands). Cells were further labeled with allophycocyanin (APC)–conjugated streptavidin (Invitrogen, Paisley, UK) and donor cells were identified using BD FACSCantoTM flow cytometer with BD FACSDiva software (BD Pharmingen, Berkshire UK). Pure populations of donor and recipient CD4 T cells (obtained from donor spleen/lymph nodes and recipient blood before transplantation, respectively) were used as positive and negative controls for donor lymphocyte identification. Positive identification of donor CD4 T cells in test samples was based on relative intensity of staining of control donor to recipient cells (Figure S1).

The human lung study received a favorable ethical opinion by the Cambridgeshire 4 Research Ethics Committee and was approval by the Health Research Authority. The study was registered with the National Institute of Health Research (NIHR) Clinical Research Network Portfolio.

### 2.2 Characterization of lymphocyte subsets released during ex vivo normothermic perfusion

Kidneys underwent 1 hour of normothermic machine perfusion, as described previously,^7^ with a leukocyte filter, RS1VAE (Haemonetics, Coventry, UK), in the circuit. After 1 hour, the filter was removed and flushed in an antegrade direction with 400 mL of sterile phosphate‐buffered saline (PBS). The filters were then incubated with 20 mL of trypsin‐ethylenediaminetetraacetic acid (EDTA) at 37°C for 10 minutes, and cells were recovered by flushing in a retrograde direction with 400 mL of sterile PBS. Cell pellets were cryopreserved with 10% DMSO (dimethyl sulfoxide) in fetal calf serum (FCS), and stored at −80°C. For flow cytometry characterization, cells were quickly thawed in Dulbecco′s Modified Eagle′s Medium (Gibco, D5030, ThermoFisher Scientific, UK) with 2% FCS and resuspended in FACS buffer (PBS, 1% FCS, 0.02% sodium azide). Cells were stained in FACS buffer for 30 minutes on ice with the following antibodies: PE anti‐human CD127 (clone eBioRDR5, ThermoFisher Scientific), Brilliant Blue 515 anti‐human CD25 (clone 2A3, BD Pharmingen), APC Cy7 anti‐human CD3 (clone SK7, BioLegend, London, UK), PE Cy7 anti‐human CD4 (clone SK3, BD Pharmingen), and dead cell exclusion dye 7‐aminoactinomycin D (BD Pharmingen). Cells were washed twice with FACS buffer after antibody staining, and cell events were collected on FACSCanto II analyzers (BD Pharmingen) and analyzed with FlowJo software (FlowJo, LLC, Ashland, OR). The human kidney study had received favorable ethical approval from Newcastle & North Tyneside 2 Research Ethics Committee REC (15/NE/0408).

### 2.3 Animals

C57BL/6J (H‐2^b^; B6) were purchased from Charles River Laboratories (Margate, UK). Bm12 mice (B6(C)‐H2‐Ab1bm12/KhEgJ [H‐2bm12]) and H‐2^b^ T cell receptor‐deficient mice (*Tcrbd* ^−/−^ [B6.129P2‐*Tcrb* ^*tm1Mom*^ *Tcrd* ^*tm1Mom*^/J]^8^) were purchased from the Jackson Laboratory (Bar Harbor, ME).

### 2.4 Heterotopic heart transplantation

Vascularized cardiac allografts were transplanted intra‐abdominally as described previously.^9^, ^10^ Heart graft survival was monitored by daily abdominal palpation, with rejection defined as cessation of a detectable beat. Grafts were excised at predetermined time points after transplantation and stored at −80°C or fixed in 10% buffered formalin. In certain experiments, recipient B6 mice were depleted of CD4 T‐regs by treatment with 0.5 mg of anti‐CD25 mAb (PC‐61, Bio X Cell, West Lebanon, NH), i.p., on day ‐1 followed by 0.25 mg, i.p., on days 1, 3, 5, and 7, in relation to bm12 heart graft transplantation. Donor T‐reg depletion was achieved by administering 0.5 mg of anti‐CD25 mAb (PC‐61), i.p., on days ‐6 and ‐2 before recovery of heart allograft. Pilot experiments confirmed that this treatment resulted in depletion of typically 85%‐90% of *FoxP3* ^*+ve*^ splenic CD4 T cells.

### 2.5 Adoptive transfer of donor/recipient–derived nT‐regs

Recipient B6 mice were adoptively transferred by tail‐vein intravenous injection with 1 × 10^6^ nT‐regs derived from B6 or bm12 animals on the first postoperative day after bm12 cardiac transplantation. nT‐regs were purified from spleens of naïve B6 or bm12 animals using the CD4^+^CD25^+^ Regulatory T Cell Isolation Kit (Miltenyi Biotec, Auburn, CA) and an autoMACS separator (Miltenyi); cell purity (typically >90% CD25^+ve^ CD4^+ve^) was analyzed by flow cytometry prior to injection.

### 2.6 Quantification of humoral autoantibody responses

Antinuclear autoantibody responses were determined by HEp‐2 indirect immunofluorescence (The Binding Site, Birmingham, UK) as described previously,^11^ by incubating test sera on slides coated with HEp‐2 cells and detecting bound antibody with FITC‐conjugated goat anti‐mouse IgG (STAR 70; Serotec, Oxford, UK). For each test serum, photomicrographs were taken, and the intensity of staining was determined by integrated morphometric analysis using MetaMorph software. The fluorescence value was then derived by comparison with a standard curve obtained for each assay by serial dilutions of a pooled hyperimmune serum that was assigned an arbitrary value of 1000 fluorescence units.

### 2.7 Histopathology

Cardiac allograft vasculopathy was assessed on elastin van Gieson –stained paraffin sections by morphometric analysis as described previously.^11^ Luminal stenosis [percentage cross‐sectional area luminal stenosis = (area within internal elastic lamina ‐ area of lumen)/area within internal elastic lamina × 100]. All elastin‐positive vessels in each section were evaluated, with approximately 10 vessels/heart analyzed.

### 2.8 Statistics

Data were presented as mean ± standard deviation (SD) where appropriate. Mann‐Whitney tests were used for analysis of nonparametric data. Two‐way analysis of variance (ANOVA) was employed for comparison of antinuclear and anti‐vimentin autoantibody responses. Graft survival was depicted using Kaplan‐Meier analysis and groups compared by log‐rank (Mantel‐Cox) testing. Analysis was conducted using GraphPad 4 (GraphPad Software, San Diego, CA). Values of *P* < .05 were considered significant.

## 3 RESULTS

### 3.1 Different CD4 T cell lineages are released from human allografts

**Figure 1:**
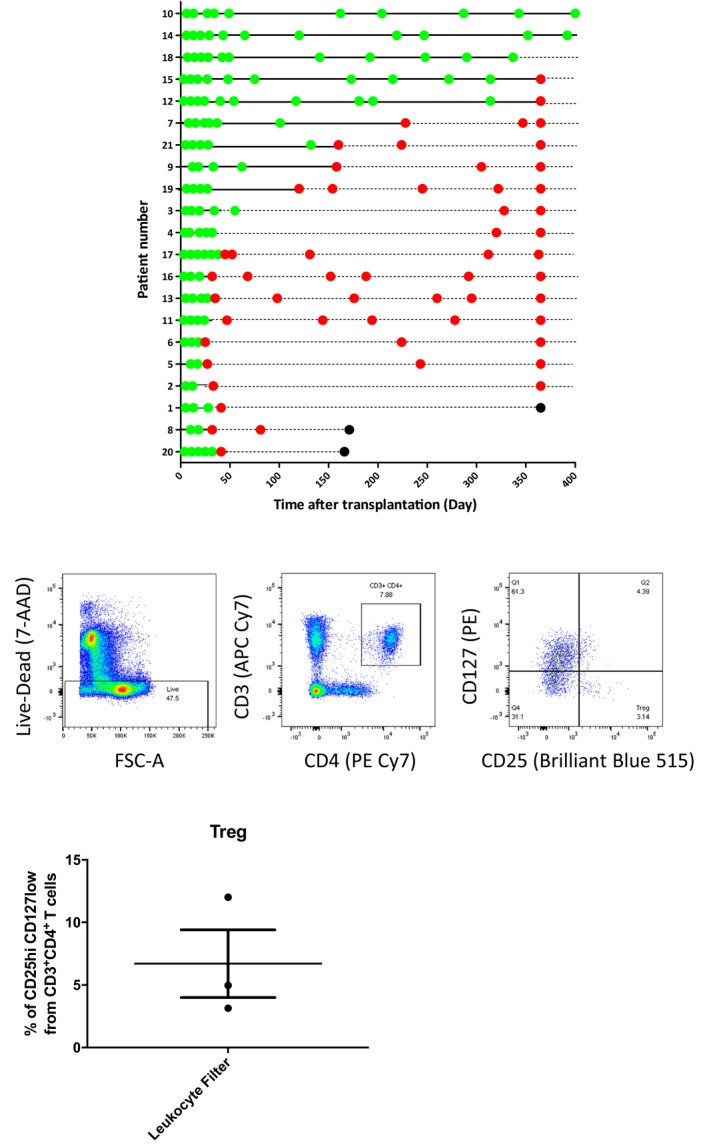
Solid organ human transplants contain passenger CD4 T lymphocyte subsets. A, Donor HLA class I mismatched antigens were used as a target for detection of donor CD4 T cell chimerism in lung transplant recipients using flow cytometry. Three patterns of donor CD4 T cell chimerism were observed: short‐term chimerism (donor CD4 T cells detectable for up to six weeks after transplantation [patients 20, 8, 1, 2, 5, 6, 11, 13, 16, 17, 3, 4 and 19]); intermediate‐term chimerism (donor CD4 T cells detectable up to 6 months after transplantation [patients 9, 21 and 7]), and long‐term chimerism (donor CD4 T cells detectable for longer than one year after transplantation [patients 12, 15, 18, 14 and 10]). Green dot, blood sample tested and donor CD4 T cells detected. Red dot, blood sample tested, donor CD4 T cells not detected. Black dot, patient died. B, Representative flow cytometry plots for analysis of live CD4 T cells recovered from leukocyte filters of human kidney organs undergoing ex vivo normothermic perfusion. Histogram depicts the proportion of CD3+ve CD4+ve T lymphocytes that expressed CD25^hi^CD127^lo^ T regulatory cell surface phenotype (n = 3)

Having previously demonstrated the presence of CD4 T effector cells within human organs recovered for transplantation,^6^, ^12^ we sought to determine whether donor CD4 T cells, and specifically, donor T‐regs, could potentially also be released into the recipient's circulation following transplantation. Human lung transplant recipients (n = 21) were therefore followed for the first year following transplantation, and the presence of circulating donor‐derived CD4 T cells determined by surface expression of mismatched HLA donor antigen (Figure S1). As shown in Figure 1, donor‐derived CD4 T cells were detectable immediately following transplantation in all patients, representing between 0.06% and 6% of the total CD4 T cell population detectable in the recipient (mean chimerism at 1 week; 1.54 ± 1.41%). Numbers of cells recovered were too small to definitely assess different T cell lineages, but real‐time polymerase chain reaction (RT‐PCR) gene expression analysis of flow sorted donor CD4 T cells (not shown) revealed profiles consistent with naïve and CD44^hi^ memory CD4 T cells, albeit samples from the same patient varied markedly at different time points, with no consistent phenotype observed. Notwithstanding, 3 different patterns of chimerism were evident (Figure 1A): transient (detectable for up to 6 weeks); intermediate (detectable for up to 6 months); or persistent (lasting for over a year).

The release of donor T‐regs was then assessed by analysis of leukocyte filters recovered from human kidneys that had been obtained using standard recovery techniques, but then perfused normothermically ex vivo using leukocyte‐depleted blood.^2^ Hence leukocytes captured by the filter in the circuit reflect those cells that would be released into the recipient circulation had the organ been transplanted without first being subject to ex vivo perfusion. CD4 T cells were readily recovered from the filters and represented 6.57 ± 1.30% of the total lymphocyte population (Figure 1B). A small, but consistently present, population of CD4 T cells with surface T‐reg phenotype (CD25^pos^CD127^lo^; 6.74 ± 4.73% of CD4 T cells) was also recovered (Figure 1B). T cells were not evident on analysis of the stored leukocyte‐depleted blood used in the circuit (not shown), suggesting that the T‐reg population had been released on reperfusion of the retrieved kidneys.

### 3.2 T‐reg depletion results in augmented humoral immunity and accelerated allograft rejection

**Figure 2:**
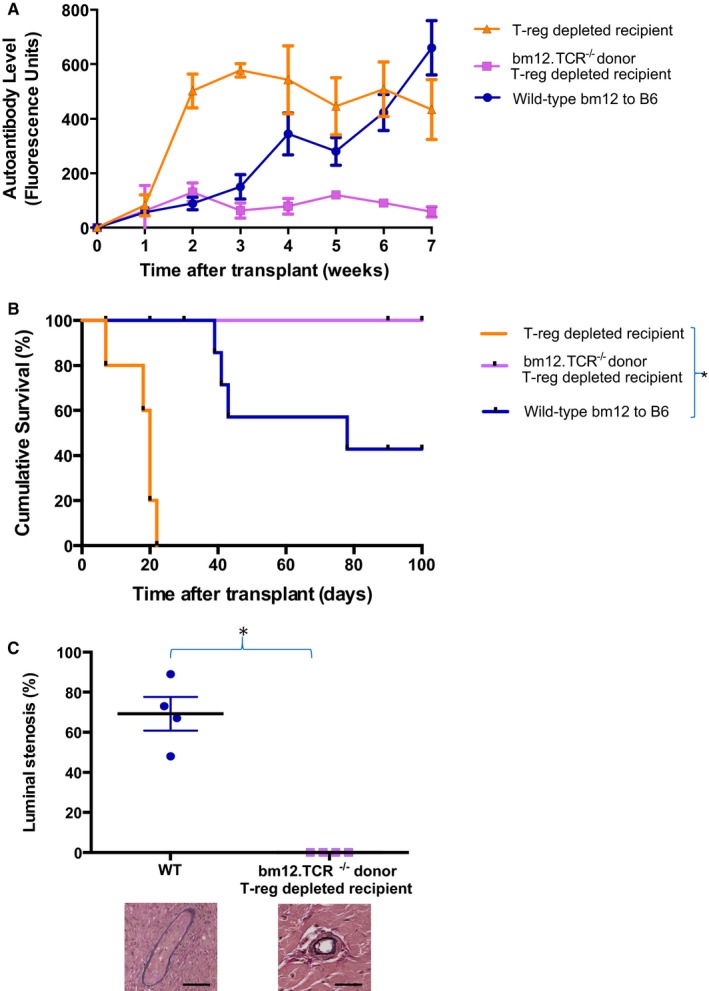
T‐reg depletion augments donor T cell–dependent effector autoantibody responses and accelerates allograft rejection. MHC‐class II mismatched cardiac allografts from WT or T cell–deficient (TCR^−/−^) bm12 donor mice were transplanted into unmodified WT C57BL/6 (B6) or T‐reg–depleted recipients and effector autoantibody responses (A), allograft survival (B), and allograft vasculopathy at explant on day 100 (C) was assessed (allograft vasculopathy for T‐reg–depleted recipients of WT bm12 heart allografts were not analyzed because of rapid graft destruction). T‐reg depletion results in augmented autoantibody responses (*P *=* *.04, Kruskal‐Wallis test) and rapid allograft rejection (*P <* *.0001, log‐rank test), but this impact is dependent on transfer of passenger donor T cells. Representative elastin van Gieson staining showing allograft vasculopathy in WT recipients compared to nondiseased vessels in (TCR^−/−^) bm12 hearts transplanted into T‐reg–depleted recipients (scale bars 100 μM). *P =* *.03, Mann‐Whitney test. Data are expressed as mean ± SD and represents a minimum of 4 animals per group

The influence of donor and recipient T‐regs on allograft outcomes was then examined using an MHC class II–mismatched murine model of chronic heart allograft rejection. Our previous work has highlighted that chronic allograft vasculopathy (CAV) in this model is associated with the development of effector autoantibody responses that are triggered by graft‐versus‐host recognition of MHC class II on host B cells by passenger donor CD4 T lymphocytes.^6^, ^12^, ^13^ In comparison to unmodified WT C57BL/6 recipients, depletion of the T‐reg population by administration of anti‐CD25 mAb to C57BL/6 mice at, and following, transplantation with bm12 (B6(C)‐H2‐Ab1bm12/KhEgJ) heart allografts resulted in much more rapid heart graft rejection, and was associated with markedly augmented host autoantibody responses (Figure 2A,B). This accelerated rejection was nevertheless dependent on adoptive transfer of donor CD4 T cells, because heart allografts from T cell–deficient bm12.TCR^−/−^ donors did not trigger host autoantibody responses and survived indefinitely, without developing CAV (Figure 2C), even following recipient T‐reg depletion (Figure 2A). This suggests that the T‐regs were principally influencing the donor T cell/host B cell axis.

### 3.3 Donor‐derived T‐regs prolong allograft survival more effectively than recipient T‐regs

**Figure 3:**
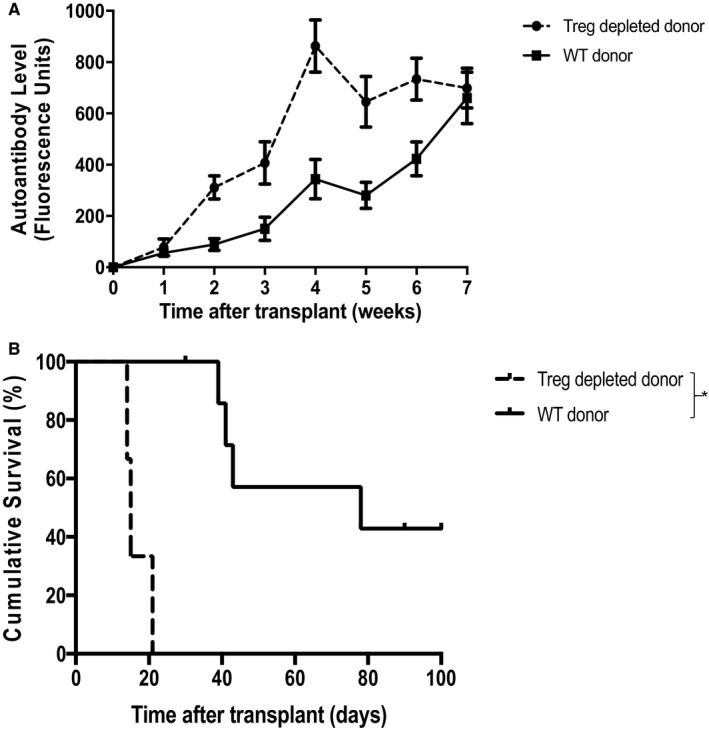
Donor T‐reg depletion results in exacerbated autoantibody production and accelerated graft loss. Heart allografts from unmodified (WT) or T‐reg–depleted bm12 donor mice were transplanted into WT C57BL/6 mice and effector autoantibody responses (A) and allograft rejection (B) were assessed. Compared to unmodified donor hearts, donor T‐reg depletion results in acute allograft rejection (median survival time [MST] 14 days vs 78 days; *P <* *.01, log‐rank test), with markedly augmented recipient autoantibody responses (**P <* *.001, 2‐way ANOVA). Data expressed as mean ± SD, n = 4

**Figure 4:**
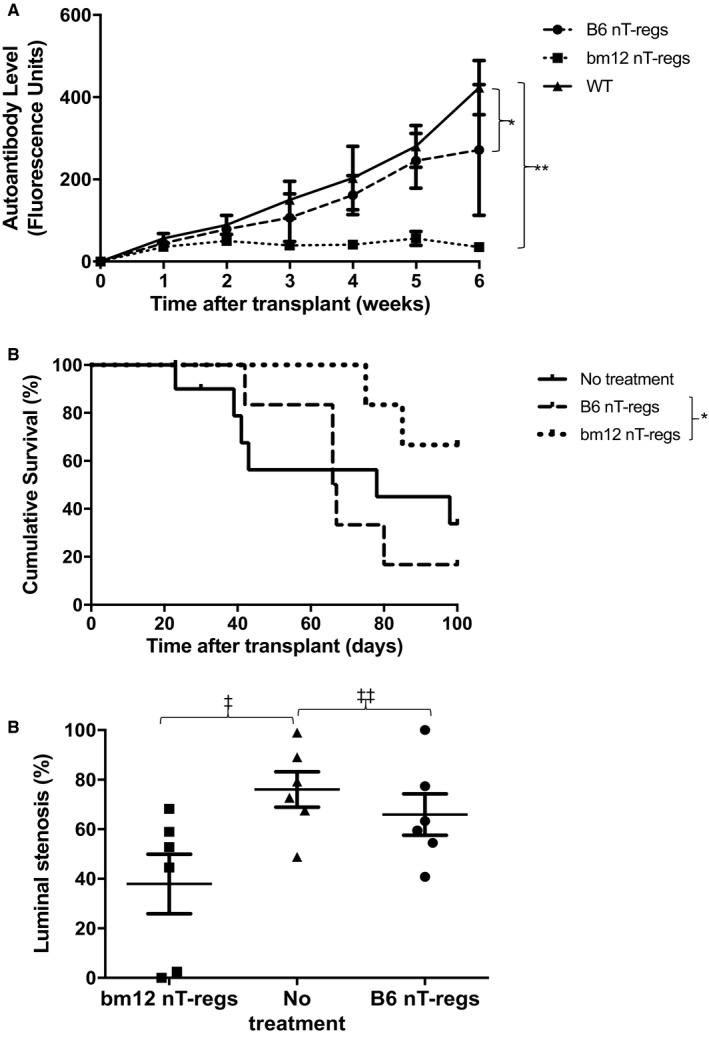
Adoptive transfer of donor nT‐reg inhibits recipient autoantibody responses and prolongs allograft survival. C57BL/6 (B6) recipients of bm12 heart allografts were adoptively transferred the day after transplantation with natural T‐regs (nT‐regs) purified from a donor (bm12) or recipient (B6) strain, and recipient autoantibody responses (A), allograft survival (B), and allograft vasculopathy (C) were assessed as detailed in Figure 2 legend. Control recipients received no treatment. Whereas administration of recipient‐strain nT‐regs had little impact on rejection responses or rejection kinetics, administration of donor‐strain nT‐regs inhibited effector autoantibody responses (*P =* *.27, **P <* *.001, 2‐way ANOVA), prolonged allograft survival (MST 91 vs 67 days; *P =* *.03. log‐rank test) and was associated with reduction in the severity of allograft vasculopathy (‡*P = *.02, ‡‡*P = *.38; Mann‐Whitney test). Data are representative of 6 animals per group, and expressed as mean ± SD, n = 6

In the preceding experiments, anti‐CD25 treatment of the recipient was continued after transplantation, raising the possibility that transferred donor T‐regs were also targeted. Notably, transplantation of heart allografts from donor bm12 mice that had received anti‐CD25 treatment before organ recovery also triggered markedly augmented autoantibody responses in WT C57BL/6 recipient mice, and heart allografts were rejected at least as rapidly as following recipient T‐reg depletion (Figure 3A,B). To test the comparative efficacy of donor versus recipient‐derived T‐regs in preventing allograft rejection, WT C57BL/6 recipients of unmodified bm12 heart grafts were additionally transferred with nT‐regs,^14^, ^15^ purified from either the recipient or donor strains. Of interest, whereas transfer of recipient‐strain nT‐regs had little discernible impact on transplant outcome, transfer of donor‐strain nT‐regs was associated with abrogation of recipient autoantibody responses, a reduction in the severity of CAV, and prolonged allograft survival (Figure 4A‐C).

## 4 DISCUSSION

Our results demonstrate that following solid organ transplantation, donor‐derived CD4 T cells are released into the recipient circulation, and, at least following lung transplantation, may persist for some time. Within a larger population of conventional CD4 T effector cells, smaller numbers of regulatory T cells can be identified, and our murine studies confirm that these can inhibit host adaptive immune responses. These findings may hold particular pertinence to ex vivo organ perfusion strategies currently being developed; they highlight that rather than blanket depletion, preservation of select passenger lymphocyte subsets within the allograft may be beneficial.

It is perhaps surprising that donor‐derived nT‐regs were more effective than recipient‐derived nT‐regs at blocking host humoral responses. Although the precise target epitopes remain ill‐defined,^16^, ^17^ nT‐regs are thought to recognize specific, self‐restricted peptide epitopes (typically autoantigens^18^). Donor‐derived nT‐regs therefore presumably recognize intact host MHC class II complexes on recipient cells via the direct pathway,^19^ and in which case, do so with a much greater precursor frequency than for a self‐restricted response, with approximately 5% of the clonal repertoire responding.^20^ We have recently demonstrated that this enables naïve donor T cells to provide promiscuous, “peptide‐degenerate” help to all host B cells, with plasma cell differentiation dictated by simultaneous B cell receptor ligation.^6^, ^21^ By extension, recognition of MHC class II alloantigen on host B cells by passenger T‐regs within the allograft is likely to provide broad inhibition of host humoral immunity. Whether this inhibition is the result of direct killing of the B cell by the T‐reg,^22^, ^23^, ^24^, ^25^ or delivery of inhibitory signals to the B cell,^26^, ^27^ or blockade of delivery of essential help from CD4 T effector cells is as yet unknown and is the subject of ongoing investigation in our laboratory. Of particular interest, our recent work has highlighted a critical role for germinal center autoantibody^28^ and alloantibody^29^, ^30^ reactions in the progression of allograft vasculopathy, and our ongoing investigations are examining the impact of donor‐derived T‐regs on host germinal center B cell/T follicular helper cell interactions.

In addition to providing support for strategies that selectively retain donor T‐regs within the allograft, our results suggest that donor‐derived T‐regs may hold potential as a cellular therapy for prolonging allograft survival. This would differ from strategies that are currently under evaluation clinically, and that typically employ recipient‐derived CD4 T‐regs that are either polyclonal or exhibit direct allospecificity for the donor.^31^ In a similar fashion to donor effector CD4 T cells (that provide promiscuous help to all B cells engaging target antigen), transferred donor‐derived T‐regs would be expected to inhibit host B cell responses against concurrently encountered alloantigen, even those alloantigens that are expressed on the T‐reg surface.^6^ Thus, it seems probable that donor‐derived T‐regs will be effective in transplant models incorporating donor‐recipient strain combinations that are more MHC‐mismatched; certainly, direct‐pathway allorecognition of host MHC class II by donor‐derived T‐regs is likely to be at least as robust in more mismatched strain combinations as in the bm12 to B6 model. For the same reasoning, we would anticipate that bm12 nT‐regs could be used as a cellular therapy to block host B cell alloresponses against a variety of different donor‐strain transplants into B6 recipients. Potency of this approach could be enhanced by either increasing the proportion of T‐regs within the transferred population that exhibit direct‐pathway allospecificity, or by first generating memory T‐regs directed against intact host MHC class II.^32^ In this regard, it is notable that heart allografts that contain memory CD4 T cells specific for host MHC class II (by priming the donor with recipient alloantigen 6 weeks prior to heart donation) are rejected much more rapidly than hearts from unmodified donors, with greatly augmented autoantibody responses.^6^, ^28^

Such a use of third‐party T‐regs to block host humoral alloimmunity would be distinctly different from proposed strategies that differentiate/expand T‐regs with self‐restricted specificity for alloantigen from the individual's endogenous T cell population,^25^, ^33^ and may offer a particular advantage. T cell help for alloantibody production can only be provided by host CD4 T cells with indirect allospecificity.^34^, ^35^, ^36^ Thus, for maximum effectiveness, recipient‐derived T‐regs would need to recognize the relevant allopeptide epitope presented by host MHC class II. Prediction of these peptides is, however, challenging, not least because the repertoire of presented allopeptide peptides may change with time.^37^ In contrast, third‐party T‐regs with direct allospecificity would be expected to interact with the individual's B cells in a peptide‐degenerate fashion, and would therefore potentially block all concurrently active B cell responses. The crucial attribute in enabling donor‐derived T‐regs to inhibit host B cell responses is avoidance of recognition and killing by host Natural Killer cells.^6^ Thus, only third‐party donors that are minimally MHC mismatched against the individual are likely to be effective. This limitation could be overcome by transduction of an individual's purified nT‐reg population with T cell receptor (*TCR*) genes,^38^ which encode direct‐pathway reactivity to that individual's own MHC class II, with the relevant *Tcr*α and *Tcr*β sequences first established by identifying dividing clones in standard mixed leukocyte reactions using third‐party cells as responders against recipient stimulators.^39^ This would generate autologous CD4 T‐regs with heightened specificity for self.

This approach may have wider uses beyond transplantation. It could, for example, be refined as a potential treatment for humoral autoimmunity, wherein nT‐regs from a third‐party donor that have direct‐pathway allospecificity for the individual's (recipient's) MHC class II antigens would be expected to block cognate interactions between autoreactive B and T helper cells in the host, thereby inhibiting autoantibody production.

## DISCLOSURE

The authors of this manuscript have no conflicts of interest to disclose as described by the *American Journal of Transplantation*.

## Supporting information


** **
