# Supplementary figures and images for: Prolongation of allograft survival by passenger donor regulatory T cells
Source: Am J Transplant. 2019 Feb 5;19(5):1371–9. doi: 10.1111/ajt.15212 (PMC6519070; doi:10.1111/ajt.15212)

Figure s1

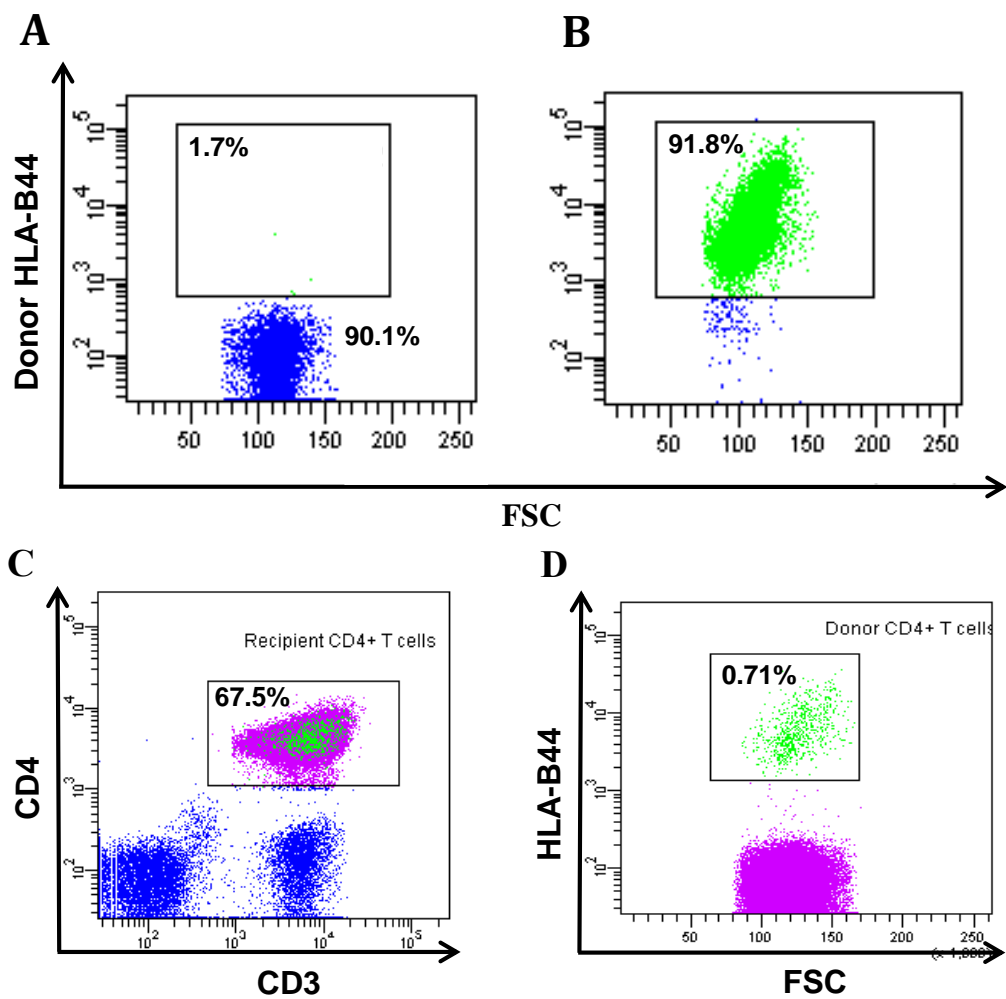

Supplement: Supplementary file 1 [file AJT-19-1371-s001.pdf]
