# Supplementary material for: Prolongation of allograft survival by passenger donor regulatory T cells
Source: Am J Transplant. 2019 Feb 5;19(5):1371–9. doi: 10.1111/ajt.15212 (PMC6519070; doi:10.1111/ajt.15212)
Supplement: Supplementary file 2 [file AJT-19-1371-s002.docx]

**Supporting Information**

**Fig. S1: Flow cytometric identification of circulating donor-derived CD4 T lymphocytes in human lung transplant recipients.**

Illustrative flow cytometry plots depicting absence of binding of anti-B44 antibody to recipient PBMCs (sampled prior to transplantation (**A**)), but with strong binding to donor splenocytes (**(B)** recovered at organ donation). Following transplantation, recipient blood was sampled, and donor-derived T lymphocytes identified within the circulating CD3^pos^CD4^pos^ T lymphocyte population **(C&D).**

**Table s1: HLA-specific antibodies for identification of donor CD4 T cell**

| **Clone-** | **HLA-Specificity** |
| --- | --- |
| BVK1F9 | B8 |
| DK7C11 | B12 |
| GV5D1 | A1/A9 (not A*24:03, A80) |
| MUS4H4 | Bw4/A24/A25/A32 |
| SN230G6 | A2/B17 |
| SN607D8 | A2/A28 |
| BVK5C4 | A9 |
| JOK3H5 | B40/B21/B13/B12/B41/B70 |
| BRO11F6 | A11/A3/A24 |
| HDG8D9 | B51/B35 |
| VTM1F11 | B27/B7/B60 |
| IND2D12 | B15/B35/B21/B70 |
| OK2F3 | A3 |
| OK6H10 | B15/B21/B56/B35/B72 |
